# Recent Advances in Cryopreservation of Animal Genetic Resources: Principles, Influencing Factors, and Material-Specific Challenges

**DOI:** 10.3390/ani16172798

**Published:** 2026-09-05

**Authors:** Tian Hua, Simin Zhao, Hao Bai, Yulin Bi, Wenming Zhao, Guobin Chang

**Affiliations:** 1 College of Animal Science and Technology, Yangzhou University, Yangzhou 225009, China; huatian0202@163.com (T.H.); smzhao333@163.com (S.Z.); ylbi@yzu.edu.cn (Y.B.); wmzhao@yzu.edu.cn (W.Z.); 2 Joint International Research Laboratory of Agriculture and Agri-Product Safety, The Ministry of Education of China, Yangzhou University, Yangzhou 225009, China; bhowen1027@yzu.edu.cn

**Keywords:** cryopreservation, cryoprotective agents, cryoinjury, germplasm conservation, vitrification, slow freezing

## Abstract

Cryopreservation plays a critical role in the long-term preservation of biological materials and has become an indispensable technology in biomedicine, agriculture, animal breeding, and biodiversity conservation. Despite substantial advances in cryobiology, the preservation of cells and tissues at ultra-low temperatures remains limited by cryoinjuries associated with ice crystal formation, osmotic stress, oxidative damage, and cellular dehydration. This review provides an overview of the historical development and fundamental principles of cryopreservation and critically summarizes the key factors affecting cryopreservation efficiency, including cryoprotective agents, antioxidants, osmotic balance, freezing and thawing protocols, and emerging ice-controlling materials. In addition, recent advances in the cryopreservation of diverse biological materials—such as sperm, oocytes, embryos, stem cells, and gonadal tissues from different animal species—are critically discussed, with particular attention to species-specific differences and the distinction between post-thaw survival and functional recovery. Finally, the current limitations of existing cryopreservation technologies and future research directions are highlighted to facilitate the development of safer and more effective preservation strategies for animal genetic resource conservation and biomedical applications.

## Simple Summary

Freezing animal cells and tissues used for reproduction and genetic resource conservation makes it possible to store valuable biological material for future breeding, research, and the protection of endangered species. However, freezing and warming can damage cells by forming ice, disturbing their water balance, and triggering harmful oxidative stress, osmotic imbalance, and membrane damage. This review explains why these injuries occur and why different species and biological materials respond differently to preservation. It brings together evidence on sperm, eggs, embryos, stem cells, cells that give rise to sperm or eggs, reproductive tissues, and selected whole-organ models. The available evidence shows that no single freezing method or protective solution is best for every material. Survival after warming does not necessarily mean that a sample can perform its intended biological function, such as fertilizing an egg, producing healthy offspring, or restoring tissue or organ function. New methods that control ice formation, deliver protective solutions more gently, or warm samples more evenly are promising, but many still require further safety testing and confirmation across laboratories. Matching preservation methods to each species and material will improve animal breeding, genetic resource banking, and wildlife conservation.

## 1. Introduction

The long-term stable preservation of biological materials is a fundamental requirement for biomedical research and biotechnological applications. Cryopreservation is widely recognized as the preferred method for achieving long-term storage of biological materials and has been increasingly applied to a diverse range of biological materials, including germline cells and reproductive tissues. Cryopreservation offers several significant advantages. It eliminates the need for continuous cell culture, thereby reducing the risks of epigenetic alterations and genetic drift associated with prolonged in vitro maintenance. In addition, it enables the preservation of desired cellular phenotypes through the establishment of cell banks. Consequently, cryopreservation has become an indispensable tool in modern biomedical laboratories for the long-term storage of valuable and rare biological resources while minimizing genetic changes that may occur during continuous culture [1]. The ability to cryopreserve oocytes, sperm, and embryos has significantly advanced assisted reproductive technologies, including in vitro fertilization (IVF), while providing an effective means for the long-term preservation of valuable germplasm resources. These advances have strengthened species conservation efforts and contributed to the maintenance of genetic diversity. In addition, cryopreservation is widely applied in modern medicine for the storage of tissues and organs intended for transplantation. It is also essential for the storage and transportation of vaccines, particularly those requiring ultra-low-temperature conditions. By maintaining the viability, functionality, and genetic integrity of biological materials, cryopreservation has become a cornerstone technology in reproductive medicine, animal genetic resource conservation, and biodiversity preservation.

Cryopreservation is a process of keeping cells in a state of suspended animation for an extended period at low temperatures, which is used to maintain the structural stability of cells [2,3]. At ultra-low temperatures, biochemical and metabolic activities within cells and tissues are greatly suppressed, allowing biological materials to enter a state of metabolic arrest and be preserved for extended periods while maintaining their structural integrity and biological function [4]. At present, two principal cryopreservation methods are widely used: slow freezing (controlled-rate freezing) and vitrification (glass formation) [5]. Based on currently published studies in different species, both slow freezing and vitrification have been shown to be effective methods for the cryopreservation of cells and tissues, although each approach exhibits distinct advantages and limitations (Table 1). Slow freezing allows precise regulation of the cooling rate, thereby minimizing various factors that may contribute to cryoinjury. However, slow freezing may also promote the formation of large extracellular ice crystals, which can cause severe mechanical damage to cells and tissues. In contrast to slow freezing [6], vitrification has emerged as another promising cryopreservation technique for living cells [7]. Vitrification enables the rapid transition of the cryopreservation solution from a liquid state to a glass-like amorphous state, thereby preventing ice crystal formation and overcoming several limitations associated with slow freezing [8]. Numerous studies have demonstrated that vitrification allows cells to rapidly enter a vitrified state, effectively reducing cryoinjury and improving post-thaw survival. This technique has been successfully applied to the cryopreservation of highly sensitive biological materials, including oocytes, ovarian tissues, and embryonic stem cells [9]. Nevertheless, vitrification requires the use of high concentrations of cryoprotective agents (CPAs), which may induce osmotic stress during CPA addition and removal procedures. Moreover, the cytotoxic effects associated with high CPA concentrations remain one of the major factors limiting the wider application of vitrification [10]. It is important to note that neither method is inherently superior; their performance depends on cell type, species, CPA formulation, and the specific protocol employed [5,6,7,8,9,10].

In recent years, advances in CPA-delivery and temperature-control technologies—including microfluidic CPA exchange, directional freezing, and laser- or magnetic-nanoparticle-based warming—have expanded the range of cells and tissues that can be cryopreserved [11,12,13,14,15]. These approaches target specific barriers, including CPA toxicity, osmotic stress, ice formation and recrystallization, spatially uneven cooling, and inadequate warming. Their performance nevertheless remains dependent on species, material dimensions, CPA formulation, and the functional endpoint assessed.

Existing reviews have generally focused on either cryobiological principles or individual material types. Few have linked biophysical mechanisms to material-specific injury across multiple cell types while consistently distinguishing post-warming survival from functional recovery. This review addresses that gap by (1) connecting fundamental cryobiology with evidence for spermatozoa, oocytes, embryos, stem and germ cells, gonadal tissues, and organ-scale models; (2) separating viability from functional endpoints such as fertilization, offspring production, germline transmission, graft function, and organ function; and (3) evaluating emerging technologies according to the strength of evidence, from physical ice control to biological function. This framework is intended to support animal genetic resource conservation and the development of safer, material-specific cryopreservation strategies. This review will provide valuable insights for the conservation of livestock and poultry genetic resources as well as future applications in reproductive biotechnology and biomedical engineering.

## 2. Literature Search and Review Scope

This article was designed as a structured narrative review to provide a critical synthesis of cryopreservation principles, major determinants of cryopreservation outcomes, and material-specific challenges in animal genetic resource conservation. It was not designed as a systematic review or meta-analysis. Relevant publications were identified through PubMed, Google Scholar, and the China National Knowledge Infrastructure (CNKI), while X-MOL was used only as a supplementary literature-discovery platform. The final search of each source was conducted on 15 September 2025.

The core PubMed search strategy was:

(“cryopreservation”[Title/Abstract] OR vitrification[Title/Abstract] OR “slow freezing”[Title/Abstract]) AND (spermatozoa[Title/Abstract] OR semen[Title/Abstract] OR oocyte*[Title/Abstract] OR embryo*[Title/Abstract] OR “stem cell”[Title/Abstract] OR “primordial germ cell”[Title/Abstract] OR gonad*[Title/Abstract] OR tissue*[Title/Abstract]) AND (animal*[Title/Abstract] OR livestock[Title/Abstract] OR poultry[Title/Abstract] OR fish[Title/Abstract] OR amphibian*[Title/Abstract] OR wildlife[Title/Abstract]).

A second, mechanism-focused PubMed search was performed using:

(“cryopreservation”[Title/Abstract] OR vitrification[Title/Abstract]) AND (“cryoprotectant toxicity”[Title/Abstract] OR “osmotic stress”[Title/Abstract] OR “oxidative stress”[Title/Abstract] OR mitochondria[Title/Abstract] OR epigenetics[Title/Abstract] OR nanowarming[Title/Abstract] OR microfluidics[Title/Abstract] OR “directional freezing”[Title/Abstract] OR “ice-recrystallization inhibition”[Title/Abstract]).

For Google Scholar, the same concepts were searched without PubMed-specific field tags. The principal searches combined the terms “cryopreservation”, “vitrification”, or “slow freezing” with terms describing the biological material (spermatozoa, semen, oocytes, embryos, stem cells, primordial germ cells, gonadal tissues, or organs) and the animal group (livestock, poultry, fish, amphibians, or wildlife). Separate searches combined cryopreservation or vitrification with each of the mechanism-related terms listed above. Because Google Scholar limits the practical length and field specificity of complex queries, these term groups were run as separate, simplified searches. X-MOL was searched using the same English keyword combinations solely to identify potentially relevant publications for verification in their original journal sources.

For CNKI, searches were conducted using corresponding Chinese-language terms. The core Chinese search combination was:

(“冷冻保存” OR “玻璃化冷冻” OR “慢速冷冻”) AND (“精子” OR “精液” OR “卵母细胞” OR “胚胎” OR “干细胞” OR “生殖细胞” OR “性腺组织”) AND (“动物” OR “家畜” OR “家禽” OR “鱼类” OR “两栖动物” OR “野生动物”).

The mechanism-focused Chinese search combination was:

(“冷冻保存” OR “玻璃化冷冻”) AND (“冷冻保护剂毒性” OR “渗透应激” OR “氧化应激” OR “线粒体” OR “表观遗传” OR “纳米复温” OR “微流控” OR “定向冷冻” OR “冰重结晶抑制”).

The reference lists of key reviews and eligible primary studies were also examined to identify additional relevant publications.

Eligible publications comprised peer-reviewed primary studies and relevant reviews addressing cryopreservation principles, factors affecting cryopreservation efficiency, or post-warming outcomes in animal spermatozoa, oocytes, embryos, stem cells, primordial germ cells, gonadal tissues, and selected organ-scale models. Human studies were considered only when they provided directly relevant mechanistic or translational information. Duplicate publications, conference abstracts, studies lacking sufficient methodological information, and articles outside the predefined scope were excluded.

Records retrieved from the different sources were first collated, and duplicates were removed by comparing titles, authors, publication years, digital object identifiers (DOIs), PubMed identifiers (PMIDs), and other available bibliographic information. One reviewer screened the titles and abstracts and subsequently assessed the full texts of potentially eligible publications. Uncertain eligibility decisions were discussed with the co-authors and resolved by consensus before final inclusion. Final inclusion was based on relevance to the biological materials, cryopreservation mechanisms, influencing factors, and post-warming endpoints covered by this review.

Because this article is a narrative review rather than a systematic review, no formal risk-of-bias assessment, meta-analysis, quantitative evidence-selection cascade, or PRISMA flow diagram was used. Evidence was synthesized qualitatively and interpreted according to the strength and biological relevance of the reported endpoints, including physical or chemical measurements, cellular viability, material-specific function and, where available, offspring production, durable graft function, or organ function.

## 3. Principles of Cryopreservation and Factors Affecting Efficiency

### 3.1. Principles of Cryopreservation

From a biological perspective, the history of cryopreservation can be traced back to the 1950s. Cryopreservation is a technique that preserves the structural integrity of cells or tissues at ultra-low temperatures, typically −80 °C or −196 °C, in the presence of cryoprotective agents (CPAs). Under these conditions, cells undergo a series of physicochemical changes that allow them to enter a state of long-term reversible metabolic arrest. The stable environment created by ultra-low temperatures can be reversed during thawing, enabling the recovery of cellular functions and the long-term preservation of biological materials [16]. Ultra-low temperatures markedly reduce cellular metabolic activity, thereby prolonging the preservation time of biological samples. In theory, when the storage temperature falls below −140 °C, biological materials can be preserved indefinitely because molecular movement and biochemical reactions are essentially arrested. Currently, biological samples are commonly stored at −80 °C for short- to medium-term preservation, whereas liquid nitrogen (−196 °C) is widely used for long-term cryopreservation [8,17].

The outcome of cryopreservation is governed by coupled heat and mass transfer. If cooling is too fast for a cell’s water permeability, water remains inside and may freeze intracellularly. If cooling is too slow, prolonged exposure to concentrated extracellular solutes can drive excessive dehydration, ionic stress, and membrane damage. The familiar inverted-U relation between cooling rate and survival is therefore material-specific, not a fixed curve for all cells [18,19]. Sample volume, container geometry, ice nucleation, and thermal gradients become increasingly important as the system expands from isolated cells to tissues and organs. Warming is equally important, as devitrification and ice recrystallization can occur when a vitrified sample spends too long in hazardous temperature ranges [20,21,22].

#### 3.1.1. Factors Affecting Cryopreservation Efficiency

##### Composition of Cryopreservation Extenders

1. Cryoprotective Agents (CPAs)

Cryoprotective agents (CPAs) protect cells through multiple interrelated but mechanistically distinct effects that should be distinguished. First, colligative effects: CPAs lower the freezing point of the solution and reduce the amount of ice formed at any given subzero temperature. This is a concentration-dependent effect common to all CPAs. Second, viscosity and vitrification effects: CPAs increase solution viscosity, which promotes glass formation during rapid cooling and suppresses ice nucleation and crystal growth. This effect is particularly important in vitrification, where high CPA concentrations are used. Third, molecular interactions: CPAs interact with water and biomacromolecules through hydrogen bonding and hydrophobic interactions. Rather than simply “disrupting” hydrogen bonds, CPAs form new hydrogen-bonding networks with water molecules, thereby reorganizing water structure and inhibiting the ordered arrangement required for ice crystallization. Non-permeating sugars such as trehalose, sucrose, and raffinose also stabilize membranes and proteins through the water-replacement hypothesis, in which sugar hydroxyl groups form hydrogen bonds with polar head groups of membrane phospholipids, maintaining membrane structure during dehydration. These three levels of action operate simultaneously but should be conceptually distinguished for rational protocol design [23,24,25]. Commonly used CPAs are generally classified into permeating and non-permeating cryoprotective agents. Both types can reduce the freezing point of the solution and the concentration of electrolytes, thereby minimizing ice crystal formation and reducing ice-induced damage to cryopreserved cells.

Permeating CPAs—such as glycerol, dimethyl sulfoxide (DMSO), propylene glycol (PG), and ethylene glycol (EG)—interact with water molecules through hydrogen bonding. This interaction lowers the freezing point of water, restricts the diffusion of water molecules during freezing, and inhibits the formation of irregular ice crystals, thereby reducing the risk of mechanically induced cellular damage [23,24]. However, the toxicity of permeating CPAs is a significant concern; their ability to penetrate cells means they can interact with intracellular molecules and subcellular structures, causing damage that is concentration- and temperature-dependent [10,26]. The statement that “permeating CPAs exhibit relatively low toxicity” is misleading without qualification—toxicity varies greatly among different CPAs, concentrations, and exposure conditions.

Non-permeating CPAs—such as hydroxyethyl starch, polyethylene glycol (PEG), polyvinylpyrrolidone (PVP), raffinose, sucrose, and trehalose—cannot penetrate the cell membrane and instead protect cells by maintaining membrane stability. These cryoprotectants increase extracellular osmotic pressure, causing intracellular water to move rapidly out of the cells during cryopreservation. This process reduces intracellular water content, thereby decreasing the formation of intracellular ice crystals and minimizing cellular damage [25].

It has been reported that CPAs affect cell membrane permeability in a concentration-dependent manner. As CPA concentration increases, membrane permeability gradually rises, facilitating the movement of intracellular water and solutes across the cell membrane. This process may lead to changes in cell volume and consequently induce osmotic stress [24,26]. Therefore, selecting an appropriate CPA concentration is essential for maintaining cellular structure during freezing and enhancing post-thaw cell survival. In practical applications, to balance the trade-off between CPA toxicity and cryopreservation efficiency, permeating CPAs are often combined with non-permeating CPAs to reduce the required concentration and toxicity of permeating CPAs while improving the cryopreservation outcomes of cells and tissues [25]. Staged loading and unloading of CPAs, guided by osmotic transport modeling, can reduce excessive volume excursions and improve downstream developmental outcomes [27]. In addition to intrinsic CPA toxicity, the processes of CPA loading and unloading themselves can cause significant injury through osmotic volume excursions. When a permeating CPA is added, cells initially shrink as water exits to balance the extracellular osmotic gradient; as the CPA penetrates the membrane, water re-enters and cells swell back toward their original volume. Upon removal (dilution), the reverse occurs: CPA exits more slowly than water enters, causing cells to swell beyond their physiological volume, potentially leading to lysis. The magnitude of these volume excursions depends on the CPA concentration, loading/unloading rate, temperature, and membrane permeability characteristics of the specific cell type [27,28]. Stepwise addition and removal, temperature-controlled exposure, and the use of non-permeating osmotic buffers can reduce osmotic shock and improve downstream developmental outcomes. These handling considerations are therefore not minor details but integral components of successful cryopreservation protocols.

2. Antioxidants

During cryopreservation, the generation of reactive oxygen species (ROS) can induce oxidative stress, leading to cellular damage or even cell death. Although cryoinjury is generally considered to result from multiple factors, excessive ROS production during the freezing and thawing processes has been recognized as a major contributor to cryopreservation-induced damage, particularly in sperm cells [29]. Freezing and thawing can alter the activity of NADPH oxidase in the plasma membrane and disrupt the mitochondrial electron transport chain, thereby promoting excessive ROS production [30,31,32]. Research has shown that excessive ROS accumulation can cause intracellular DNA damage, lipid peroxidation of polyunsaturated fatty acids, and amino acid oxidation, particularly oxidative damage induced by hydrogen peroxide generated within mitochondria.

**Figure 1:**
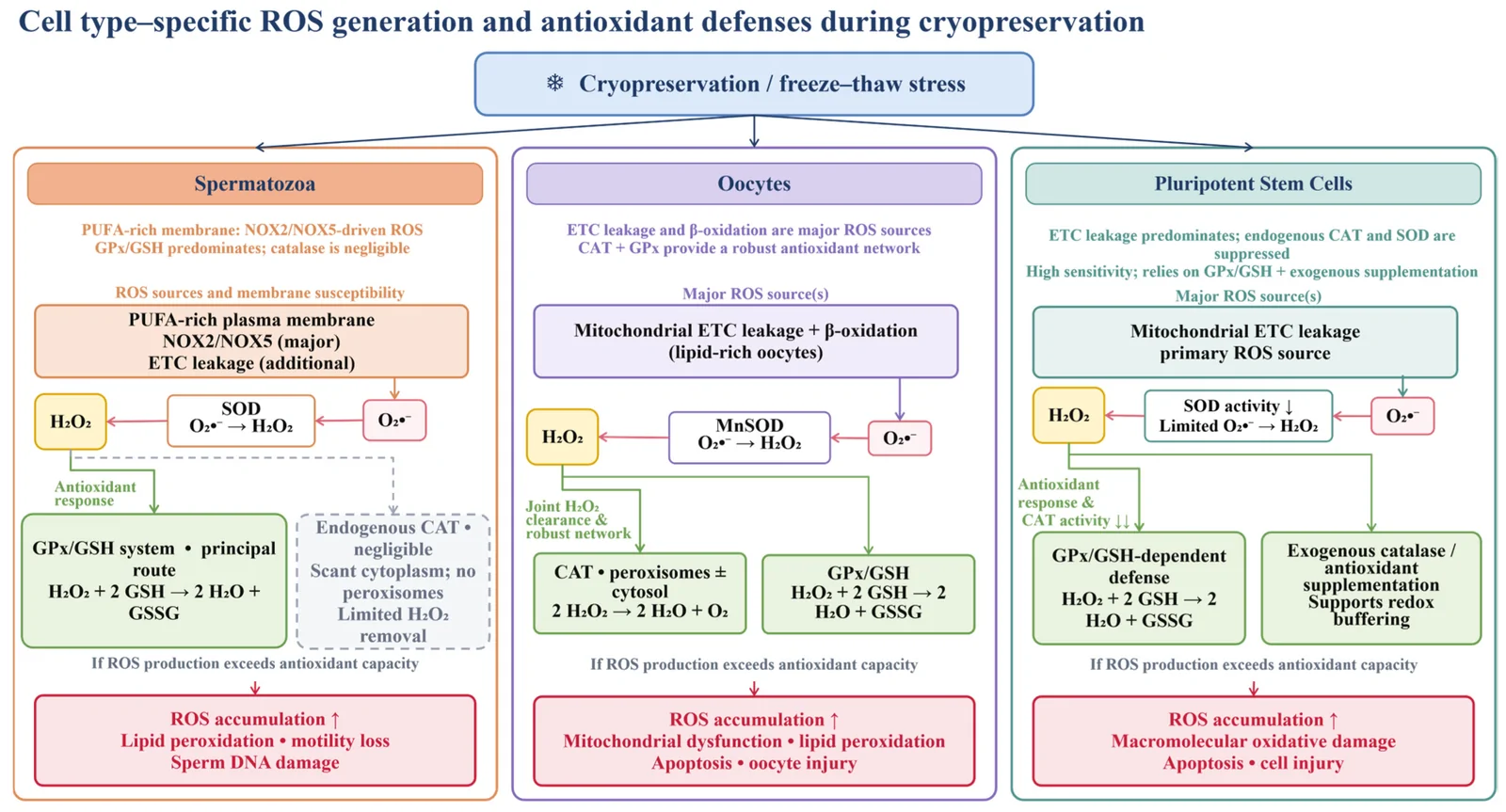
Schematic comparison of cell type-specific ROS generation and antioxidant defense systems in spermatozoa, oocytes, and pluripotent stem cells during cryopreservation/freeze–thaw stress. Abbreviations: ROS, reactive oxygen species; ETC, electron transport chain; NOX2 and NOX5, NADPH oxidases 2 and 5, respectively; NADPH, reduced nicotinamide adenine dinucleotide phosphate; PUFA, polyunsaturated fatty acid; O_2_, oxygen; O_2_•^−^, superoxide; SOD, superoxide dismutase; MnSOD, manganese superoxide dismutase; H_2_O_2_, hydrogen peroxide; CAT, catalase; GPx, glutathione peroxidase; GSH, reduced glutathione; GSSG, oxidized glutathione; DNA, deoxyribonucleic acid. Solid arrows indicate the depicted ROS-generation and antioxidant-defense pathways, whereas dashed arrows and outlines denote the negligible contribution of endogenous CAT in spermatozoa; ↑ indicates ROS accumulation, and ↓ and ↓↓ indicate reduced and markedly reduced enzyme activity, respectively.

To mitigate the detrimental effects of oxidative stress on cells and tissues, antioxidants are commonly incorporated into cryopreservation media. Antioxidants can be broadly classified into two categories. The first category comprises enzymatic antioxidants, also known as endogenous antioxidant enzymes, including glutathione peroxidase (GPx), glutathione reductase (GR), superoxide dismutase (SOD), and catalase, all of which are involved in the natural antioxidant defense system. The glutathione system operates as a functionally coupled redox cycle that is critical for maintaining cellular redox balance during cryopreservation. Glutathione peroxidase (GPx) reduces hydrogen peroxide and lipid peroxides to water and alcohols, respectively, using reduced glutathione (GSH) as the electron donor. In this reaction, GSH is oxidized to glutathione disulfide (GSSG). Glutathione reductase (GR) then regenerates GSH from GSSG using NADPH as a cofactor, completing the cycle. The depletion of GSH or NADPH pools—which can occur during cryopreservation due to oxidative stress and metabolic disruption—impairs this cycle, making cells more vulnerable to oxidative damage [33,34,35]. Therefore, the efficacy of antioxidant supplementation should be evaluated not only by the concentration of exogenous antioxidants but also by the availability of GSH and NADPH within the endogenous redox system. Saleem [36] reported that semen cryopreservation significantly reduces the antioxidant capacity of sperm. During the freezing–thawing process, the depletion of antioxidant reserves in seminal plasma weakens the antioxidant defense system of sperm. The resulting imbalance between oxidants and antioxidants promotes excessive ROS production, leading to oxidative stress-induced damage. This not only impairs sperm motility but also activates autophagy, ultimately resulting in programmed cell death, including apoptosis and necrosis, and may even trigger premature sperm capacitation (Figure 1).

It is important to note, however, that the response to antioxidant supplementation is often nonlinear and context-dependent. For example, in buffalo spermatozoa, MitoQ showed a concentration-dependent window in which a low dose was beneficial and higher concentrations were detrimental [37]. Similarly, ascorbate can also promote radical chemistry in the presence of redox-active iron; this chemical possibility must be considered in metal-containing media [38]. Therefore, supplementation with antioxidants during cryopreservation requires careful optimization of concentration and timing. Perumal et al. [39] reported that glutathione supplementation during bull sperm cryopreservation improved post-warming sperm motility and acrosomal integrity, thereby increasing conception rates. Limaye et al. [40] reported that supplementation of the cryopreservation medium with 200 μmol of a vitamin E analogue effectively improved the post-thaw viability of human hematopoietic stem cells (HSCs) while maintaining their biological characteristics. Numerous studies have demonstrated that oocyte cryopreservation can induce mitochondrial oxidative stress, as well as lipid and protein peroxidation within mitochondria, thereby promoting the release of various apoptotic factors and ultimately leading to oocyte apoptosis. Therefore, the addition of exogenous antioxidants, such as melatonin and resveratrol, to oocyte cryopreservation media can reduce oxidative damage-induced apoptosis by directly or indirectly scavenging ROS [35].

In summary, although the addition of antioxidants during cryopreservation can significantly improve cryopreservation outcomes, the total antioxidant capacity of cells and tissues should be carefully considered in practical applications. Furthermore, excessive concentrations of antioxidants may adversely affect cell growth and function. Therefore, the use of an appropriate antioxidant concentration is essential to achieve optimal cryopreservation efficiency.

3. Osmotic Pressure of the Freezing Medium

Cells have limited tolerance to culture media with different osmotic pressures, and previous studies have reported the physiological osmotic limits of various cell types. Excessive hyperosmolarity or hypoosmolarity can alter cell membrane permeability, integrity, and function, and in severe cases, may disrupt membrane structure, resulting in irreversible cellular damage or even cell death. Controlled cellular dehydration is critical during cryopreservation because it restricts excessive intracellular ice crystal formation. However, excessive dehydration may cause cell shrinkage and ultimately compromise cell viability [41]. Therefore, changes in osmotic pressure represent a critical factor in cryopreservation, as they regulate the flux of water across the cell membrane during equilibration, freezing, and thawing [42,43].

Some researchers have reported that an osmotic pressure of 285–295 mOsm/kg is optimal for buffalo semen cryopreservation. Within this osmotic range, most sperm maintain normal morphology, motility, and DNA integrity. In contrast, excessively high osmotic pressure can cause sperm dehydration, resulting in irreversible cellular damage [44]. Wei et al. [45] reported that a high-osmolarity environment is more suitable for mouse semen cryopreservation. Compared with sperm collected in a medium with an osmolarity of 290 mOsm/kg, sperm collected in a medium with an osmolarity of 415 mOsm/kg exhibited higher fertilization capacity during in vitro artificial insemination. In contrast to other cell types, oocytes are more susceptible to cryodamage because of their unique structural characteristics and therefore require more stringent cryopreservation conditions. Mullen et al. [28] investigated the morphological changes in oocyte spindles under different osmotic conditions and found that both hyperosmotic and hypoosmotic environments could induce abnormal spindle morphology. They further reported that cryopreservation of human oocytes at an osmotic pressure of 295 mOsm/kg increased the number of viable cells after thawing.

In conclusion, selecting an appropriate osmotic pressure is essential for maintaining cellular status and improving cell viability and integrity following cryopreservation and thawing. Furthermore, understanding the osmotic tolerance limits of different cell types is critical for the development of optimal cryopreservation protocols.

4. Cryoprotective Material

Based on the above analysis of the factors affecting cryopreservation efficiency, effective control of ice formation within cells and tissues is particularly important for reducing freezing-induced damage and improving cryopreservation outcomes. With the rapid development of materials science and chemistry, a variety of novel materials with ice-controlling properties have been developed and widely applied as ice inhibitors during cryopreservation, providing new opportunities for enhancing cryopreservation efficiency.

For example, antifreeze proteins (AFPs) possess ice recrystallization inhibition (IRI) activity, which enables them to effectively suppress ice crystal growth and reduce cryoinjury [46]. Ice recrystallization is the process by which small ice crystals gradually grow into larger crystals at subzero temperatures. This phenomenon is commonly observed during the thawing of cryopreserved cells or tissues. Studies have shown that ice recrystallization can lead to cellular or tissue dehydration, as well as structural and functional damage. AFPs have a strong affinity for ice crystal surfaces, and the preferential binding between AFP molecules and ice crystals inhibits further ice crystal growth [47]. Lee et al. [48] cryopreserved mouse ovaries treated with AFPs and found that the addition of AFPs helped maintain a greater number of intact follicles. Although numerous studies have demonstrated the beneficial effects of AFPs on cryopreservation, excessive use of AFPs may induce genetic alterations in cryopreserved samples, thereby limiting their widespread application in the field. Moreover, the immunogenicity and potential toxicity of AFPs in mammalian systems remain concerns [49]. The discovery of synthetic polymers and nanomaterials has led to their introduction as emerging ice inhibitors, which also show considerable potential for cryopreservation. For example, poly(vinyl alcohol) (PVA) exhibits ice recrystallization inhibition (IRI) activity. Compared with AFPs, PVA demonstrates a more pronounced IRI effect. Naullage et al. reported that a lower degree of polymerization helps maintain the high IRI activity of PVA. In addition, graphene oxide (GO), which possesses structural features similar to those of natural AFPs, has been widely investigated in equine semen cryopreservation. The addition of only 0.01 wt% GO increased the cryopreservation efficiency of horse sperm from 24.3% to 71.3% [50]. However, graphene oxide also raises dispersion, dose, residue, and reproducibility questions [49,50]. Evidence from physical ice assays should therefore be followed by cell-specific function and safety testing.

Beyond the materials described above, many other novel ice-inhibiting materials—including synthetic ice-recrystallization inhibitors, polymers such as poly(vinyl alcohol), and various nanomaterials—have been developed and successfully applied in cryopreservation, leading to significant advances in the field [46,47,50,51,52]. The discovery and application of these materials have greatly promoted the development of cryobiology and provided new opportunities for medicine, reproductive biology, and animal breeding.

##### Temperature Changes During Freezing and Thawing

Exposure of cells to low temperatures is often detrimental to cell survival. To better understand the effects of low temperatures on cryopreservation, it is important to recognize that changes in cellular structure are closely associated with temperature fluctuations. When the temperature falls below 0 °C, intracellular water begins to freeze. Since water typically accounts for approximately 80% of tissue mass, the cooling rate during cryopreservation is a critical determinant of cell survival. In the early 1960s, Mazur [18] demonstrated that the rate of temperature change during cryopreservation is important because it regulates water transport across the cell membrane and thereby indirectly influences the probability of intracellular ice formation. For example, during sperm cryopreservation, the freezing rate affects post-thaw sperm motility and viability. If the freezing rate is too slow, sperm are exposed to a hypertonic environment, which accelerates cellular dehydration and increases the risk of cell death. In addition, it may result in incomplete penetration of cryoprotective agents throughout the sperm cell, leading to cryodamage [53]. Several studies have reported that a freezing rate of 15–60 °C/min is optimal for mammalian semen cryopreservation and is associated with improved post-thaw survival [54]. Therefore, selecting an appropriate cooling rate is essential to minimize cryoinjury during the cryopreservation of cells and tissues. An excessively rapid cooling rate can lead to intracellular ice formation, whereas an excessively slow cooling rate may expose cells to lethal solution effects [55]. It is important to note that the optimal cooling rate varies among different cell types. This is because different cells differ in their capacity to transport water across the plasma membrane. Therefore, the cooling rate should be adjusted according to the membrane permeability characteristics of each cell type.

**Figure 2:**
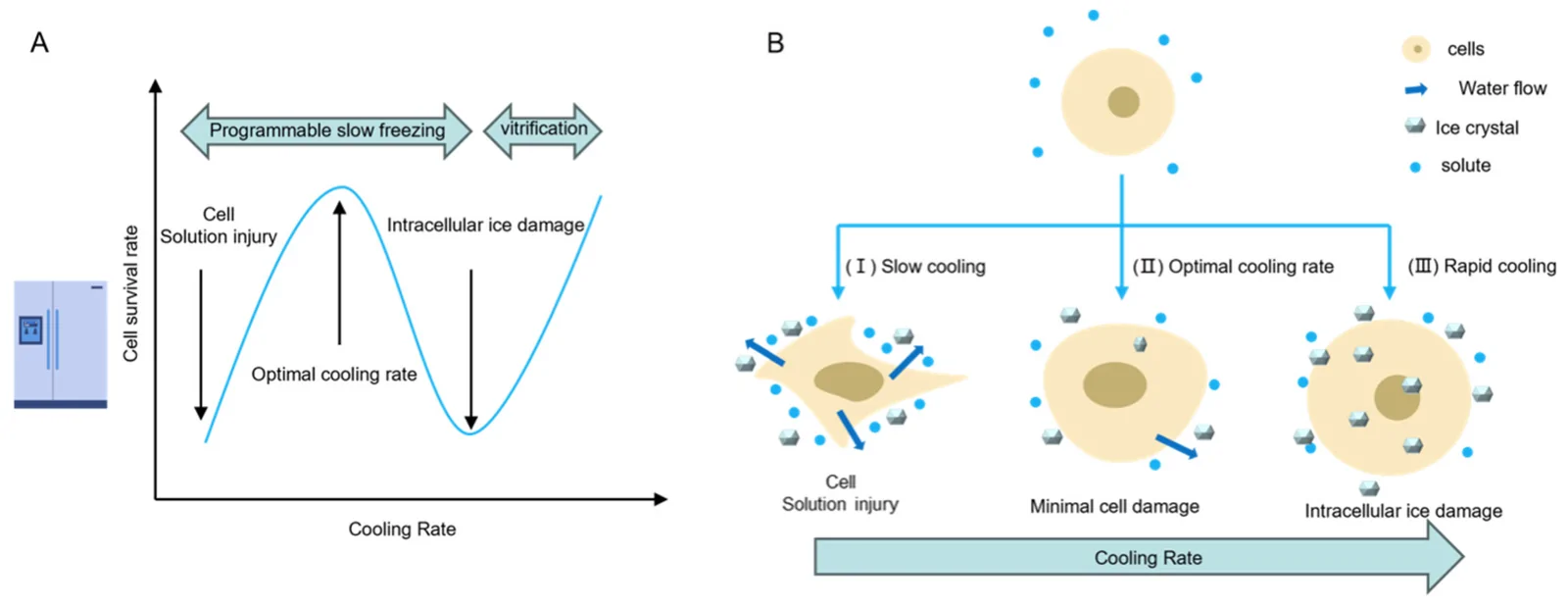
(**A**) Schematic diagram of the relationship between cooling rate and cellular responses during cryopreservation. (**B**) Small blue arrows indicate water efflux, blue dots represent water molecules, gray symbols represent ice crystals, and the horizontal arrow indicates increasing cooling rate.

It is generally accepted that rapid warming during thawing is essential to prevent ice recrystallization [20], The theoretical basis for this view is rooted in thermodynamics. During the freezing–thawing process, there is an intermediate critical temperature range (−15 to −60 °C) that is highly detrimental to cells and can readily cause cellular damage or even cell death. Cells must pass through this temperature range twice: once during cooling and again during warming. The warming rate during thawing also significantly affects the cryopreservation outcome of cells and tissues. Numerous studies have shown that passage through the critical temperature zone (−15 to −60 °C) during thawing promotes the conversion of small ice crystals and residual liquid water into larger ice crystals. In particular, slow warming facilitates ice recrystallization, coalescence, and crystal growth, all of which have been shown to cause cellular damage or even cell death (Figure 2). Therefore, inhibiting ice crystal formation and growth during thawing is critical for improving cryopreservation efficiency and remains a major focus of cryobiology research. In contrast, rapid warming minimizes ice recrystallization, allowing only the melting of ice within cells and tissues. Current recommendations for post-thaw cell recovery suggest transporting cryopreserved samples in liquid nitrogen vapor (rather than on ice or dry ice) and subsequently placing them in a 37 °C water bath for 90–120 s to maximize post-thaw survival [21,22].

Although the optimal cooling and warming conditions may vary among different cell types, the effectiveness of cryopreservation depends largely on the interaction between cooling and warming rates. Therefore, the rate of temperature change during the freezing–thawing process is a critical determinant of cell survival. At present, elucidating the mechanisms of intracellular ice formation and mitigating the associated cellular damage remain major research priorities in the field of cryobiology.

## 4. Cryopreservation of Different Genetic Materials Across Species

Due to interspecies differences in physiological characteristics, the physicochemical responses to low-temperature conditions can vary substantially among different cell types and species. Therefore, species-specific physiological and biological characteristics should be carefully considered during cryopreservation to maximize the post-thaw survival and functional integrity of each type of genetic material [19]. The following sections summarize recent progress in the cryopreservation of sperm, oocytes, stem cells, and gonadal tissues, together with the major factors and considerations affecting their cryopreservation outcomes.

### 4.1. Sperm

Throughout this review, semen refers to the collected fluid matrix containing spermatozoa and seminal plasma, whereas sperm or spermatozoa refers to the cells being evaluated. Cryopreservation protocols are usually applied to spermatozoa suspended in semen or artificial extenders; therefore, the term used should reflect whether the matrix or the cell is being discussed.

Currently, cryopreservation is widely applied in animal artificial insemination (AI) and in vitro fertilization (IVF), and the technology continues to advance and mature [56]. It is important to distinguish between semen (the collected fluid matrix) and spermatozoa (the cells being evaluated), as this distinction matters because seminal plasma, extender dilution, and cell concentration can change cryoresponse. Sperm cryopreservation is mature enough for routine artificial insemination in several livestock species, yet a protocol that works for bovine sperm cannot be transferred directly to poultry, fish, or wildlife. Membrane lipid composition, head morphology, osmotic tolerance, seminal plasma, and packaging format all influence the optimal CPA and cooling rate [56,57,58,59,60,61,62,63].

Santiago-Moreno et al. [57] reported that, in chicken sperm cryopreservation, a two-step freezing protocol using a moderate cooling rate (from 5 °C to −35 °C at 7 °C/min, followed by cooling from −35 °C to −140 °C at 60 °C/min) resulted in relatively high sperm motility, acrosome integrity, sperm motion quality, and plasma membrane integrity. Under these conditions, the fertilization rate reached approximately 80–90%, thereby maximizing post-thaw sperm survival [58]. In practical bovine sperm cryopreservation, the slow-freezing method is commonly employed. Specifically, a programmable freezing system with cooling rates of −0.25 °C/min and −0.5 °C/min is considered optimal for bovine semen cryopreservation, as it can achieve relatively high post-thaw sperm viability [59]. There are also slight differences in sperm cryopreservation among different species. Buffalo sperm are more susceptible to cryodamage than bovine sperm during the freezing process. Currently, two main cryopreservation protocols are used for buffalo semen. The first is a stepwise cooling protocol, in which semen straws are maintained at 5 °C for 4 min, 15 °C for 7 min, −80 °C for 15 min, and −130 °C for 15 min before being plunged into liquid nitrogen. The second is a continuous cooling protocol, in which the temperature is reduced from 5 °C to −30 °C and subsequently from −30 °C to −100 °C at predetermined cooling rates. The samples are then held at −100 °C for 5 min before immersion in liquid nitrogen. Both protocols have been shown to achieve satisfactory cryopreservation outcomes with high post-thaw sperm viability. However, the stepwise cooling protocol generally results in a slightly higher recovery rate than the continuous cooling protocol [60,61].

Byrne et al. [62] compared the effects of two freezing rates, 5 °C/min (rapid) and −0.5 °C/min (slow), on the fertilization rate of sheep sperm. The cleavage rate (57% vs. 26%) and blastocyst rate per oocyte (28% vs. 13%) of rapidly frozen semen were higher than those of slowly frozen semen. For thawing sperm samples, it is recommended to thaw them at a rate of 43.5 °C/min on a dry surface or at a rate of 55.2 °C/min in 35 °C air to achieve optimal sperm survival rate and post-thaw motility, though these rates were optimized for human sperm and may not apply directly to all species [64]. Maintaining sperm motility and acrosome integrity is essential for achieving high fertilization rates during artificial insemination.

Across species, post-thaw sperm function is influenced by cooling and warming rates, membrane composition, extender formulation, and packaging conditions; therefore, no protocol should be transferred between species without validation. Evidence from chickens, cattle, buffaloes, and sheep demonstrates substantial species-specific differences in optimal thermal profiles and functional outcomes [57,58,59,60,61,62,63,64]. Spermatozoa are particularly vulnerable to oxidative injury because their limited cytoplasmic volume provides relatively low antioxidant capacity and their polyunsaturated-fatty-acid-rich membranes are susceptible to lipid peroxidation. During cryopreservation, mitochondrial electron leakage and membrane-associated NOX pathways contribute to ROS formation, whereas the GPx/GSH system represents a major pathway for H_2_O_2_ detoxification and CAT activity is generally very low [29,30,31,32,33,34,36]. Antioxidant supplementation should therefore be optimized according to species, formulation, and dose. Common forms of cryoinjury in frozen–thawed sperm include reduced acrosome integrity, decreased motility, impaired fertilization capacity, and lower overall survival rates. Nevertheless, continuous improvements in cryopreservation protocols have significantly reduced these adverse effects and enhanced post-thaw sperm viability and fertilization potential.

For wildlife conservation, sperm cryopreservation has produced F_2_ offspring in an amphibian model [65]. Nevertheless, applications in endangered species remain constrained by scarce samples and incomplete knowledge of reproductive biology, requiring case-specific protocol development [66,67,68].

### 4.2. Oocytes

In modern animal husbandry and reproductive medicine, oocytes represent an important resource for preserving fertility. Oocyte cryopreservation plays a critical role in basic research, genetic resource conservation, and clinical applications by enabling the long-term storage of female gametes in gene banks. However, compared with other cell types, oocytes are characterized by their large size, high water content, and unique intracellular structures, which present considerable challenges for cryopreservation. Oocyte developmental stage is an important determinant of cryopreservation outcome. Immature oocytes lack a fully assembled metaphase-II spindle and may therefore avoid some forms of spindle injury that affect mature oocytes; however, immature-stage cryopreservation does not eliminate osmotic, mitochondrial, or structural damage [69,70]. Cryopreservation can alter spindle integrity, cortical granule distribution, mitochondrial function, and redox balance, potentially reducing subsequent fertilization and developmental competence [28,35,69,70]. The magnitude of these effects varies with species, donor age, health status, and reproductive history along with oocyte stage, CPA formulation, and cooling and warming protocol. Accordingly, post-warming morphology or survival should be reported separately from maturation, fertilization, blastocyst development, and offspring outcomes (Table 2).

Oocytes are particularly challenging to cryopreserve because their large size, abundant mitochondria, high lipid content, and specialized cytoskeletal structures create multiple targets for cryoinjury. Cryopreservation-induced mitochondrial dysfunction can increase ROS generation, disrupt the meiotic spindle and cortical-granule distribution, and impair fertilization and subsequent embryonic development. Although oocytes possess substantial cytoplasmic antioxidant capacity, their redox response varies with species and developmental stage. Melatonin and resveratrol have shown protective effects in some protocols, but their efficacy is concentration- and context-dependent [35,77]. Therefore, protocol optimization should be based on functional endpoints rather than morphology or survival alone.

### 4.3. Embryos

Embryo cryopreservation is a critically important component of assisted reproductive technologies (ART) and genetic resource banking. The first successful vitrification of mouse embryos was reported by Rall and Fahy in 1985, demonstrating that ice-free cryopreservation could achieve high post-warming survival [8]. Since then, embryo cryopreservation has been applied across multiple mammalian species, but with marked variation in success. Developmental stage changes blastomere number, intercellular junctions, cavity formation, lipid content, and permeability; therefore, cleavage-stage embryos and blastocysts should not be grouped without qualification. Vitrification often performs well in small mammalian embryos, such as mouse and bovine blastocysts, when CPA exposure and warming are tightly controlled [5,6,8,9,25]. Vitrification can support post-warming development and pregnancy in cattle when CPA exposure and warming are tightly controlled; however, reported pregnancy rates vary with embryo stage, embryo-production system, transfer protocol, and recipient factors. Pregnancy and live-birth outcomes should therefore be supported by directly corresponding primary studies and reported separately from post-warming survival.

Functional endpoints for embryo cryopreservation should include re-expansion, hatching, implantation, pregnancy, birth, and offspring health, as appropriate. Recent technological developments have aimed to improve the achievement of these endpoints by reducing CPA-related damage during loading and unloading. A microfluidic embryo-on-chip platform provided gradual CPA loading and reduced handling-associated stress in a mouse model [12]. In contrast to the progress made in mammalian embryos, vitrification of fish embryos remains extremely difficult. The principal obstacles include large yolk volume, which limits CPA penetration; the presence of a chorion that restricts solute exchange; and compartmentalized water distribution within the embryo, which promotes heterogeneous ice formation [14]. Laser nanowarming improved zebrafish embryo recovery in a proof-of-concept study, but only a small fraction progressed to later outcomes [14]. The evidence therefore supports targeted technology development, not a general claim that embryo cryopreservation has been solved across taxa. For livestock species, embryo cryopreservation is most advanced in cattle and sheep, where both slow freezing and vitrification are used commercially; for poultry and fish; however, embryo preservation remains largely experimental due to species-specific structural barriers. This remains an active area of research requiring species-specific optimization.

### 4.4. Stem Cells, Primordial Germ Cells, and Spermatogonial Stem Cells

Stem cells contribute to regenerative medicine, toxicology, and animal genetic-resource conservation, but these applications involve biologically distinct cell populations [78,79,80,81,82,83,84]. HSCs, MSCs, ESCs, SSCs, and PGCs should therefore not be evaluated using a single post-warming endpoint. Appropriate measures include colony formation or engraftment for HSCs, immunomodulatory function and differentiation for MSCs, pluripotency and genomic stability for ESCs, spermatogenesis for SSCs, and germline transmission or offspring production for PGCs [78,82,83,84,85,86,87,88,89,90,91].

Conventional slow cooling with DMSO is widely used for HSCs and MSCs, whereas pluripotent stem cells often require protocols that limit dissociation-induced apoptosis and oxidative injury [85,86,87]. ESCs may be particularly susceptible to mitochondrial dysfunction, ROS-mediated DNA damage, and altered pluripotency, whereas MSCs and HSCs may possess more robust antioxidant capacity [83,85,86,87]. PGC responses remain species dependent; in chickens, controlled-rate freezing with 10% EG preserved survival and characteristic properties, and cryopreserved PGCs can retain germline competence [88,89,90].

Protocol optimization should therefore integrate cryopreservation with post-warming culture conditions and assess cell-type-specific function, genetic stability, and long-term biological performance rather than viability alone.

### 4.5. Gonadal Tissue

The testis and ovary contain spermatogonial stem cells and oocytes, respectively, both of which exist in a state of developmental arrest. Ovarian cortex contains primordial, primary, and more advanced follicles; it does not simply store “primary oocytes” in isolation. Testicular tissue contains spermatogonia, somatic support cells, extracellular matrix, and vascular structures. Tissue thickness creates CPA and temperature gradients, so a viable surface layer can coexist with central injury [92,93,94,95,96,97,98,99]. Therefore, the cryopreservation of gonadal tissues, followed by fertility restoration through surgical transplantation after thawing, represents a viable strategy for the conservation of genetic resources. Numerous studies have demonstrated that gonadal tissues can be stored long-term under cryogenic conditions. Compared with isolated cells, both slow-freezing and vitrification protocols have shown favorable outcomes for gonadal tissue preservation, enabling the recovery of a greater number of functional gametes following thawing and transplantation [91]. Vitrification is widely used for certain tissues (e.g., ovarian cortex and small tissue pieces), whereas slow freezing remains common for larger tissue blocks requiring deeper CPA penetration. However, the stringent requirements associated with surgical procedures remain one of the major challenges limiting the broader application of this technology.

Using poultry as a model, Song et al. further developed a simple and efficient cryopreservation protocol [92]. They used DMSO as the CPA and applied a controlled cooling procedure (−1 °C/min to −80 °C) for the cryopreservation of testicular tissue. Using this protocol, offspring were produced from cryopreserved testicular tissue for the first time. Higaki et al. found that the use of 1.3 M dimethyl sulfoxide for the cryopreservation of zebrafish testicular tissue resulted in the highest spermatogonial cell viability (60%) [93]. Spermatogonial cells derived from both fresh and cryopreserved testicular tissues retained their ability to colonize the recipient gonads following transplantation into wild-type zebrafish. Furthermore, the recipient fish were able to produce donor-derived offspring through natural mating [94]. For the cryopreservation of human testicular tissue, the use of 1.5 M DMSO and 0.15 M sucrose as cryoprotectants, combined with a slow-freezing protocol at a cooling rate of −1 °C/min, has been shown to be an effective approach. Following thawing, a relatively high viability of spermatogonial stem cells can be maintained [95]. The successful cryopreservation of testicular tissue and the generation of spermatogonial cells significantly increase the chances of successfully restoring fertility.

Ovarian tissue cryopreservation preserves primordial follicles within the ovarian cortex together with their surrounding somatic and stromal microenvironment. Their small size, low metabolic activity, and early developmental stage may contribute to relative cryotolerance [96], making ovarian cortical tissue a promising target for female germplasm banking. In sheep, cryopreservation with 2 M DMSO and slow cooling at 2 °C/min resulted in a follicular mortality rate of 16.0% ± 1.0% [97]. Human ovarian tissue is commonly preserved using DMSO-based slow freezing with controlled cooling and ice seeding, although interlaboratory variation precludes a universal protocol [98] In mice, vitrification using EG, DMSO, serum, and sucrose has produced favorable follicle-survival outcomes [99]. Protocol selection should therefore consider tissue thickness, CPA penetration, species, and the intended functional endpoint. Unlike isolated-cell preservation, tissue cryopreservation retains the follicular microenvironment but requires more complex post-warming culture, transplantation, or in vitro follicle-growth procedures.

### 4.6. Whole Organs

Whole organs combine every major cryobiological challenge: long diffusion distances, vascular and parenchymal compartments, thermal gradients, fracture risk, CPA toxicity, and the need for immediate integrated function after rewarming. For that reason, histology or isolated-cell viability is insufficient. The rat-kidney nanowarming study provides unusually strong evidence because rewarmed organs were transplanted and supported recipient survival [13]. It remains a small-animal proof of concept; larger organs will require validated CPA perfusion, nanoparticle distribution and clearance, uniform heating, vascular protection, sterility, and long-term safety.

## 5. Evaluation of Cryopreservation Outcomes

Cryopreservation should be evaluated as a chain rather than as a single viability test.

### 5.1. Physical and Chemical Validation

Physical measurements confirm whether the intended cooling, glass formation, ice control, and warming were achieved. Relevant reporting variables include material dimensions and cell concentration, extender composition, cryoprotective agent (CPA) identity and molarity, loading and unloading steps, exposure temperature and time, nucleation method, cooling and warming profiles, and carrier geometry.

### 5.2. Cellular and Molecular Assessment

Cellular assays assess membrane integrity, osmotic recovery, apoptosis or necrosis, reactive oxygen species (ROS) generation, mitochondrial function, DNA damage, and cell-specific structures. Molecular and epigenetic measurements are also valuable when interpreted cautiously. Changes in ROS, mitochondrial potential, gene expression, or DNA methylation may be transient, cell-selective, or caused by handling and culture rather than storage itself. Cross-sectional differences do not demonstrate heritable genetic alteration. Studies should therefore include fresh-handled controls, exposure-only controls, matched culture time, biological replication, and, where relevant, longitudinal or offspring follow-up [58,77,100,101,102].

Increasing evidence suggests that cryopreservation may induce epigenetic modifications, oxidative stress, mitochondrial dysfunction, and altered gene expression after thawing. Cryopreservation can change mitochondrial bioenergetics and redox state [101]. Studies of DNA methylation have reported small, locus-dependent changes in rainbow trout [100] and broader oxidative and methylation effects under a specific Tibetan pig cryopreservation protocol [102]. These findings support protocol-specific surveillance but do not establish inevitable heritable changes.

### 5.3. Material-Specific Functional Assessment

The next level of evaluation determines whether the cryopreserved material retains its intended biological function. For spermatozoa, relevant outcomes include motility, acrosome and plasma membrane integrity, mitochondrial and DNA integrity, and fertilizing capacity [58,63]. For oocytes, post-warming morphology and survival should be reported separately from maturation, fertilization, blastocyst development, and offspring outcomes [28,35,69,70]. Functional endpoints for embryos include re-expansion, hatching, implantation, pregnancy, birth, and offspring health, as appropriate. For stem cells, primordial germ cells (PGCs), and spermatogonial stem cells (SSCs), the selected endpoints should reflect their intended uses, including colony formation, differentiation, engraftment, germline transmission, or offspring production [78,82,83,84,85,86,87,88,89,90,91]. For gonadal tissues and whole organs, cellular viability alone is insufficient; evaluation should consider fertility restoration, tissue reconstruction, transplantation outcomes, or integrated organ function [13,91,92,93,94,95,96,97,98,99].

### 5.4. Long-Term Biological Outcomes and Reporting Standards

The final level of evaluation, when ethically and technically feasible, includes offspring production, durable graft function, or organ function. The long-term effectiveness of cryopreservation has been extensively investigated, particularly with regard to storage duration and the possibility of genetic abnormalities. Available studies demonstrate that some cells and tissues can be preserved for extended periods and recover their biological functions after thawing without detectable genetic alterations.

**Figure 3:**
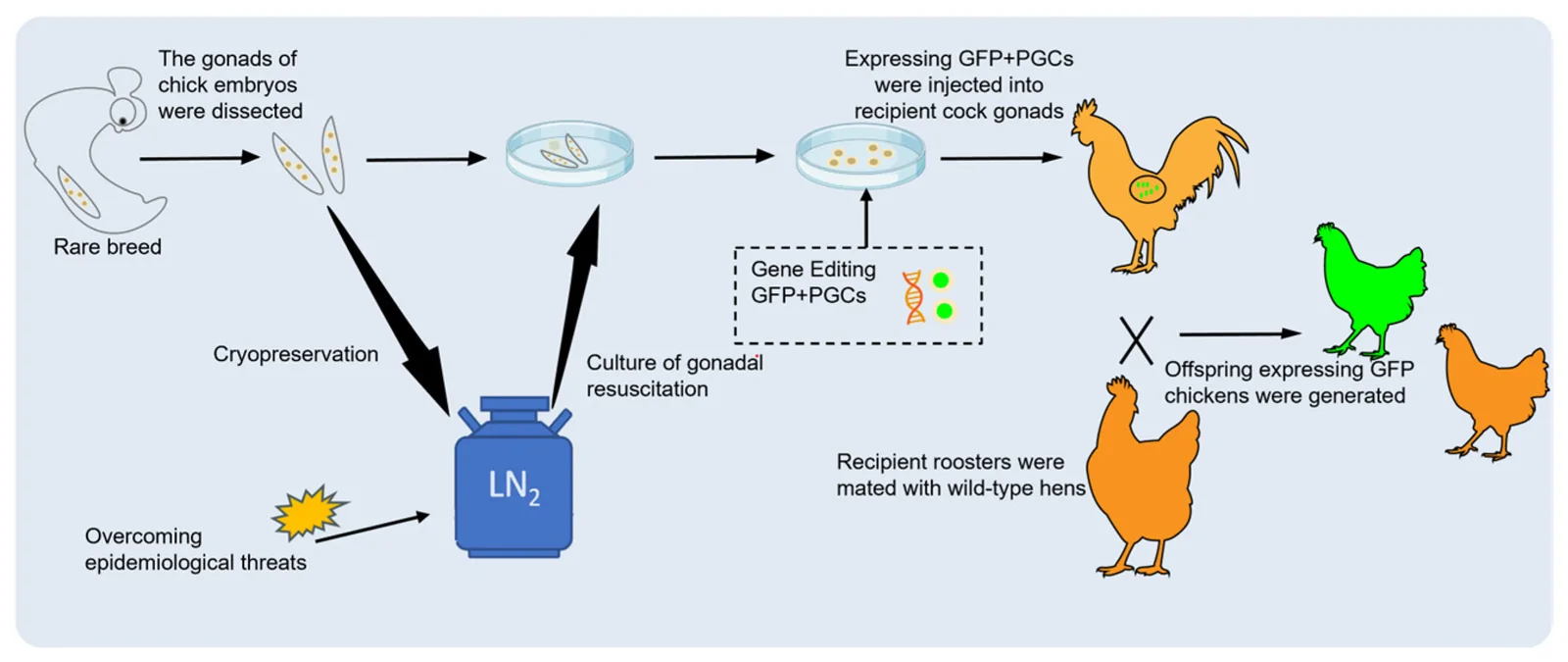
Schematic diagram of rare-breed restoration through gonadal cryopreservation. Arrows indicate the experimental workflow, the dashed box denotes the gene-editing step, the multiplication sign (×) indicates mating, the yellow burst-shaped symbol represents epidemiological threats, and the green chicken icon represents GFP-expressing offspring. LN_2_, liquid nitrogen; GFP, green fluorescent protein; PGCs, primordial germ cells.

Wu et al. reported that the cryopreservation of testicular cells from mice, rats, rabbits, and baboons for more than 14 years preserved SSC viability [103]. Following thawing and transplantation, these cells colonized the seminiferous tubules of recipient testes. Mouse and rat SSCs also re-established complete spermatogenesis, and the resulting sperm produced fertile offspring without detectable genetic or epigenetic abnormalities. Similarly, cryopreserved Drosophila PGCs generated F_1_ offspring after transplantation and established germline stem cell populations in recipient gonads [104]. Chicken PGCs cryopreserved for 5 months produced chimeric offspring after transplantation into recipient embryos [105]. Mating between male and female chimeras subsequently produced homozygous offspring derived from the cryopreserved–thawed PGCs, with normal reproductive performance (Figure 3). Cryopreserved adult quail testicular cells differentiated into functional gametes in recipient gonads and generated donor-derived chimeric offspring [106]. Pregnancy and offspring production were also achieved after the transplantation of cryopreserved ovarian tissue into irradiated recipient mice [107].

Although long-term cryopreservation appears safe based on the currently available evidence, the absence of detectable genetic alterations in the reported cases should not be interpreted as proof that such alterations never occur. Reporting should include species and breed or strain, age or developmental stage, storage duration, and clearly defined endpoints. Without complete methodological and outcome information, apparently conflicting studies cannot be reconciled and promising protocols cannot be reproduced.

## 6. Prospects

Future research should prioritize the material-specific integration of low-toxicity CPA formulations, controlled CPA delivery, ice management, and rapid uniform warming. Microfluidic platforms can standardize CPA loading and unloading for small cells and embryos [11,12]; synthetic ice-recrystallization inhibitors may reduce reliance on conventional CPAs [51,52]; directional freezing may improve spatial ice control in thick tissues [15]; and nanoparticle-assisted warming may reduce thermal gradients in larger specimens [13,14]. However, these approaches remain at different stages of validation and require standardized assessment of cytotoxicity, residues, reproductive safety, scalability, and batch reproducibility.

Translation should be judged according to intended biological function rather than post-warming survival alone. Whole tissues and organs require validated perfusion, uniform CPA and nanoparticle distribution and removal, fracture control, sterility, and long-term graft testing. In contrast, wildlife applications require low-input protocols compatible with scarce samples and recovery routes such as surrogate germline transplantation, in vitro gametogenesis, or assisted fertilization [65,89,91,93,94,104]. Interlaboratory reference protocols, predefined functional endpoints, and reporting of both positive and negative findings will be essential for improving reproducibility and practical translation.

## 7. Conclusions

Cryopreservation is a key technology for the long-term preservation of animal genetic materials and is widely applied in animal breeding, reproductive biotechnology, and biodiversity conservation. Its efficiency is mainly limited by cryoinjury caused by ice crystal formation, osmotic imbalance, oxidative stress, and cryoprotectant toxicity. The success of cryopreservation largely depends on the coordinated optimization of cryoprotective systems, temperature-control procedures, and species-specific biological characteristics. Slow freezing and vitrification can both work when matched to the material; neither has universally low injury, low apoptosis, or superior efficiency. The strongest protocols link physical control to cell-specific function and, where possible, fertility, offspring, graft, or organ outcomes. Although recent advances in vitrification and cryoprotective systems have significantly improved post-thaw survival and functional recovery of cells and tissues, the preservation of highly sensitive materials, such as oocytes and embryonic stem cells, remains challenging. Emerging microfluidic, ice-inhibiting, directional-freezing, and nanowarming tools expand what may be preserved, but their value depends on reproducible delivery, dose and safety limits, scale, and functional validation. A species- and material-specific framework is therefore the most reliable route from promising laboratory survival to usable animal genetic-resource banking.

## Figures and Tables

**Table 1:** Conditional comparison of controlled-rate freezing and vitrification.

Cryopreservation Method	CPA Concentration	Cost and Time	Cryoinjury Pattern	Application Scope
Slow Freezing	Generally lower	Depends on equipment, carrier, and scale; may be higher or lower than vitrification depending on protocol	Intracellular and extracellular ice crystal formation may occur	Suitable for many cell types; well-established for sperm, blood cells, and some tissues
Vitrification	Generally higher	Depends on equipment, carrier, and scale; may be higher or lower than slow freezing depending on protocol	Suppresses ice formation when vitrification and rapid warming are successful; devitrification and ice recrystallization may occur if thermal control is inadequate.	Suitable for scarce and precious cells such as oocytes, embryos, and some stem cells

Note: Neither controlled-rate freezing nor vitrification is universally superior; outcomes depend on the species, biological material, CPA formulation, carrier geometry, and cooling and warming protocol [5,6,7,8,9,10].

**Table 2:** Cryopreservation of oocytes from different species.

Species	Cryopreservation Protocol	Post-Warming Survival	Developmental or Functional Outcome	Reporting Limitation
Goat	Solid-surface vitrification (SSV): 35% ethylene glycol + 5% PVP + 4% trehalose	~60% in vitro survival [71]	Not reported	Oocyte stage, denominator/N, and time point NR
Cattle	Vitrification: 15% EG + 15% PG + 0.5 M sucrose	≥90% survival (authors’ data) [72]	Blastocyst: 7.4% ± 4.1% (EG + PG) vs. 1.7% ± 3.0% (EG + DMSO) [72]	Denominator/N and assessment time point NR
Mice	Vitrification: 1.0 M DMSO	93% morphological survival [69]	Maturation 74% vs. 83% fresh; fertilization 70% vs. 81% fresh	Denominator/N NR; fresh comparator reported
Pig	Vitrification: 17.5% EG + 17.5% PG + 0.3 M sucrose, 30 s	97.4% [73,74]	27.7% blastocyst formation after IVF [73]	Confirm oocyte stage, denominator/N, and time point
Sheep	Vitrification: 17.5% EG + 17.5% PG + 0.3 M sucrose, 30 s	Effective preservation [75]	Not reported	The qualitative phrase ‘effective preservation’ was removed
Human	Vitrification: 15% EG+ 15% DMSO + 0.5 M sucrose + 12% SSS, staged exposure	81% [70,76].	Not reported	Confirm oocyte stage, denominator/N, and source type

Note: Outcomes are shown separately and should not be pooled across studies. NR, not reported in the cited source or not extractable from the current manuscript; EG, ethylene glycol; PG, propylene glycol; DMSO, dimethyl sulfoxide; PVP, polyvinylpyrrolidone; SSS, serum substitute supplement; IVF, in vitro fertilization.

## Data Availability

No new data were created or analyzed in this study. Data sharing is not applicable to this article.

## Abbreviations

The following abbreviations are used in this manuscript:

Abbreviation	Definition
AI	artificial insemination
AFPs	antifreeze proteins
CPA	cryoprotective agent
DMSO	dimethyl sulfoxide
EG	ethylene glycol
ESCs	embryonic stem cells
GO	graphene oxide
GPx	glutathione peroxidase
GR	glutathione reductase
GSH	glutathione (reduced)
HSCs	hematopoietic stem cells
IRI	ice recrystallization inhibition
IVF	in vitro fertilization
MSCs	mesenchymal stem cells
PGCs	primordial germ cells
PG	propylene glycol
PVA	poly(vinyl alcohol)
ROS	reactive oxygen species
SSCs	spermatogonial stem cells
SOD	superoxide dismutase
